# The New Antimicrobial Peptide SpHyastatin from the Mud Crab *Scylla paramamosain* with Multiple Antimicrobial Mechanisms and High Effect on Bacterial Infection

**DOI:** 10.3389/fmicb.2016.01140

**Published:** 2016-07-21

**Authors:** Zhongguo Shan, Kexin Zhu, Hui Peng, Bei Chen, Jie Liu, Fangyi Chen, Xiaowan Ma, Shuping Wang, Kun Qiao, Kejian Wang

**Affiliations:** ^1^State Key Laboratory of Marine Environmental Science, College of Ocean & Earth Science, Xiamen UniversityXiamen, China; ^2^Fujian Collaborative Innovation Center for Exploitation and Utilization of Marine Biological Resources, Xiamen UniversityXiamen, China; ^3^State-Province Joint Engineering Laboratory of Marine Bioproducts and Technology, Xiamen UniversityXiamen, China

**Keywords:** SpHyastatin, *Scylla paramamosain*, antimicrobial peptide, antimicrobial mechanism, membrane permeabilization, immune protection

## Abstract

SpHyastatin was first identified as a new cationic antimicrobial peptide in hemocytes of the mud crab *Scylla paramamosain.* Based on the amino acid sequences deduced, it was predicted that this peptide was composed of two different functional domains, a proline-rich domain (PRD) and a cysteine-rich domain (CRD). The recombinant product of SpHyastatin displayed potent antimicrobial activities against the human pathogen *Staphylococcus aureu*s and the aquatic animal pathogens *Aeromonas hydrophila* and *Pseudomonas fluorescens*. Compared with the CRD of SpHyastatin, the PRD presented better antimicrobial and chitin binding activities, but both regions were essential for allowing SpHyastatin complete antimicrobial activity. The binding properties of SpHyastatin to different microbial surface molecules suggested that this might be an initial and crucial step for performing its antimicrobial activities. Evaluated using propidium iodide uptake assays and scanning electron microscopy images, the antimicrobial mechanism of SpHyastatin was found to be prone to disrupt cell membrane integrity. Interestingly, SpHyastatin exerted its role specifically on the surface of *S. aureu*s and *Pichia pastoris* whereas it directly killed *P. fluorescens* through simultaneous targeting the membrane and the cytoplasm, indicating that SpHyastatin could use different antimicrobial mechanisms to kill different species of microbes. As expected, the recombinant SpHyastatin increased the survival rate of crabs challenged with *Vibrio parahaemolyticus*. In addition, SpHyastatin could modulate some *V. parahaemolyticus*-responsive genes in *S. paramamosain*.

## Introduction

Antimicrobial peptides are widespread in microbes, plants and animals and they serve as a first line of defense against pathogen invasion (Lay and Anderson, 2005; Hancock and Sahl, 2006; Goyal et al., 2013; Sun et al., 2014; Wang et al., 2015). Most of them are cationic and amphipathic, but vary extremely in length, structural property and antimicrobial activity spectra. To successfully develop and effectively utilize the recombinant or the synthetic product of a new AMP requires a clear understanding of its antimicrobial characterization including the mechanisms and functions. Although several forms of antimicrobial mechanisms of AMPs are known, it is generally accepted that most AMPs are prone to inhibit or kill varieties of pathogens by directly altering or disrupting their cell membranes. The representatives are magainin 2, protonectin and CPF-C1, which are shown to kill bacteria by inducing membrane permeabilization (Jang et al., 2012; Wang et al., 2013; Xie et al., 2014). In addition, some AMPs, such as buforin II and Bac7, appear to penetrate the cell membrane and exert antimicrobial activity by damaging critical intracellular targets including nucleic acids, proteins and even the ribosomes without lysing the cell membrane (Bustillo et al., 2014; Mardirossian et al., 2014; Guida et al., 2015). Interestingly, a recent study states that tachyplesin I may act via different action modes to kill *Escherichia coli* and *Staphylococcus aureus* (Hong et al., 2015), suggesting that some AMPs might have more than one active method of attack against different species of microbes. AMPs possess multi-functionality except for their direct antimicrobial activities (Lai and Gallo, 2009; Smith and Dyrynda, 2015). They can also modulate host gene expression, function in chemotaxis, induce/inhibit cytokine production, etc. (Mookherjee et al., 2006; Lai and Gallo, 2009). For instance, expression of an AMP, msrA3, in potato not only provides resistance against *Fusarium solani* but also mitigates plant defense responses and alters plant development (Goyal et al., 2013). Shrimp anti-lipopolysaccharide factor suppresses the production of proinflammatory cytokine in LPS-stimulated cervical cancer HeLa cells, implying its anti-inflammatory effects on mammalian hosts (Lin et al., 2013). *Penaeus monodon* penaeidin acts as a cytokine that can promote granulocyte and semi-granulocyte adhesion (Li et al., 2010), and participates in the wound-induced inflammation response in *P. monodon* (Li and Song, 2010). Thus, the multiple immunomodulatory reactions of AMPs are thought of as potential enhancement of host defense against invading infectious agents.

Structure-function associated studies show that some AMPs have their specific antimicrobial domains, for instance, the active histone regions including buforin I and parasin I are usually derived from the N-terminal portion of histone (Kim et al., 2000; Cho et al., 2002). Conversely, the activity of hepcidin relies on its C-terminal CRD which is derived from the preprohepcidin through propeptide convertase cleavage (Xu et al., 2008; Wang et al., 2009). Nevertheless, a recently reported defensin identified in Japanese horseshoe crab shows its activity in both the N-terminal hydrophobic region and the cationic C-terminal region, which are active against Gram-positive and -negative strains, respectively (Kouno et al., 2008). In addition, some truncated fragments of AMPs, such as Bac7 (1–23), PR-39 (1–22), and arasin 1 (1–23), exert a very potent antimicrobial activity in comparison with their entire peptides (Paulsen et al., 2013; Veldhuizen et al., 2014; Guida et al., 2015). Therefore, study of the structure-function association of an AMP would facilitate clearly elucidating its antimicrobial mechanism.

Compared with *in vitro* investigation, *in vivo* functional and mechanism studies of AMPs at molecular level are relatively less studied. The difficulty is partly due to the actual effective concentration of AMPs being much lower when measured in physiological conditions than *in vitro* (Lai and Gallo, 2009). *In vivo* efficacy and safety profiles of some AMPs are commonly evaluated in mice in which, for example, the roles of VR3, an arginine- and valine-rich-hairpin-like AMP, and pardaxin in response to pathogen infection are well investigated in different mouse models (Dong et al., 2012; Huang et al., 2014). Recently, artificial challenge experiments using live pathogens are also used in marine animals for evaluating the efficacy of an AMP *in vivo*. The antimicrobial effects of LcLEAP-2A and LcLEAP-2C are determined in large yellow croaker with the challenge of *Vibrio alginolyticus* (Li et al., 2014). In addition, the antiviral activity of mytilin derived fragments is evaluated in *Litopenaeus japonicus* and *Palaemon serratus* injected with white spot syndrome virus (WSSV) (Roch et al., 2008), as well as a study on epinecidin-1 carried out in zebrafish infected with *Vibrio vulnificus* (Pan et al., 2011).

In recent years, marine-derived bioactive peptides have attracted much attention for exploitation because of their potential development of new antimicrobial agents. AMPs are an important finding in this field. To date, more than 2,000 AMPs of different origins have been reported in the AMP database (APD^1^) (Pushpanathan et al., 2013), but only several AMPs have been identified in crabs since the first AMP characterized in *Carcinus maenas* (Schnapp et al., 1996). In our previous study, a new *hyastatin*-like gene (*SpHyastatin*) encoding 131 amino acids was screened from a subtractive suppression hybridization cDNA library of *Scylla paramamosain* (Chen et al., 2010). SpHyastatin showed a certain sequence homology with the reported hyastatin which is an AMP identified in *Hyas araneus* (Sperstad et al., 2009). The study on SpHyastatin greatly attracted our attention to reveal its characteristics and biological functions because of its potential importance in immune defense. *S. paramamosain* possesses vital economic value, and is one of the most important marine breeding crabs in China. Normally, *S. paramamosain* is prone to suffering from pathogen infection, especially bacterial infection which often causes high mortality to the crabs. However, no effective immune control measure has been established as yet. Therefore, clarifying the new AMP SpHyastatin of *S. paramamosain* would provide us with more immune knowledge on how this peptide exerts a role against pathogenic invasion when the crabs are raised in a complex marine environment. In our study, SpHyastatin was successfully expressed in *Pichia pastoris* and its antimicrobial activities against both aquatic animal and human pathogens were determined *in vitro*. Following MIC assay, confocal laser scanning microscopy and SEM assays were employed to further differentiate and evaluate its antimicrobial features on different species of pathogens *in vitro*. In addition, the immune protective and immunomodulatory functions in *S. paramamosain* conferred by SpHyastatin were further investigated.

## Materials and Methods

### Animals and Strains

All animal procedures were carried out in strict compliance with the National Institute of Health Guidelines for the Care and Use of Laboratory Animals and were approved by the animal welfare and ethics committee of Xiamen University. Microbial strains used in this study were listed in Supplementary Table S1. *P. pastoris* GS115 was purchased from Invitrogen (USA). Other strains were purchased from the China General Microbiological Culture Collection Center.

### Recombinant Expression of SpHyastatin and Proline-Rich Domain SpHyastatin in the Yeast *P. pastoris*

The recombinant product of SpHyastatin was generated by cleaving the fusion protein scygonadin/SpHyastatin, expressed in the yeast *P. pastoris*. The fragment inserted into the *Pichia* expression vector pPIC9K (Invitrogen USA) consisted of the mature peptide sequence of *scygonadin* (FJ769142), a flexible peptide linker (Gly-Gly-Pro-Gly-Ser-Gly), an enterokinase recognition site (Asp-Asp-Asp-Asp-Lys), and the mature peptide sequence of *SpHyastatin* (JX228177). The sequences encoding either mature scygonadin or SpHyastatin were amplified using either a pair of primers SCY-F/SCY-R or SpHyas-F/SpHyas-R as listed in Supplementary Table S2. Then, the two PCR products were used as templates to amplify a complete *scygonadin*/*SpHyastatin* covalently linked sequence using primers SCY-F and SpHyas-R. The resulting recombinant plasmid pPIC9K-*scygonadin*/*SpHyastatin* was transformed into *E. coli* DH5α and verified by sequencing. 3 μg of the recombination DNA plasmid of linearization by SacI was used to transform *P. pastoris* cells using electroporation. A positive clone was chosen for large-scale recombinant expression induced with 0.5% methanol. The subsequent supernatant, containing the secreted fusion peptide, was collected and dialyzed in PBS (phosphate-buffered saline, pH8.0) before being purified using immobilized metal affinity chromatography. The fusion protein was cleaved using recombinant enterokinase (Novagen, USA) in the ratio of 1 U/50 μg at 16°C for 12 h. The second purification for the target protein, SpHyastatin, was accomplished with immobilized metal affinity chromatography. Finally, the collected SpHyastatin was dialyzed in Milli-Q water and stored at -80°C for later use.

Specific primers SCY-F paired with SCY-R and SpHyas-N term (Supplementary Table S2) paired with SpHyas-R were used to amplify the cDNA fragments of *scygonadin* and PRD *SpHyastatin* (the region from Tyr 1 to Arg 80 of the mature peptide) before a *scygonadin*/PRD *SpHyastatin* covalently linked sequence was obtained using primers SCY-F and SpHyas-R. The recombinant PRD was expressed and purified using a procedure similar to the one used for expression and purification of the recombinant SpHyastatin.

### Peptide Synthesis of the CRD

CRD SpHyastatin consisted of 35 amino acids (3.8 kDa) as follows: SNCWARCPGYPNGDSLCCRQYGACCSTSYPVPYKG. The chemically synthesized CRD SpHyastatin was purified via reverse phase HPLC by the Invitrogen Trading Co., Ltd., Shanghai, China. The purity of the synthetic peptide was determined to be over 95%. The peptide was dissolved in sterile Milli-Q water and stored at -80°C until use.

### Antimicrobial Assays

A variety of microbial strains were used to test the antimicrobial activities of the recombinant SpHyastatin, the PRD and the synthetic CRD. Mueller–Hinton broth, Difco marine broth and YPG medium were used for culturing the standard bacterial strains, the marine bacteria and the yeasts, respectively. The MIC and MBC (minimum bactericidal concentration) values were determined in triplicate on separate occasions using liquid growth inhibition assays (Bulet et al., 1993; Wang et al., 2009). Mid-logarithmic phase cultures of microbes were diluted in 10 mM NaPB (pH 7.2–7.4), and then approximately 10^4^ CFU/well for bacteria and 10^3^ CFU/well for yeast were incubated with serially diluted peptide in the presence of the corresponding broth in a 96 well flat-bottom tissue-culture plate. Cultures were grown in the dark for 24 or 48 h at 28°C without shaking. The lowest protein concentration induced no visible growth when compared with the negative control, and was defined as the MIC value. Cultures with no visible growth were then placed on appropriate media. The MBC was calculated as the concentration that prevented growth of more than 99.9% of microorganisms after incubation for 24 h at 28 or 37°C.

### Bactericidal Assay

Kill-curve studies, performed using *S. aureus*, were conducted as previously described (Wang et al., 2009). The concentration corresponding to 4 × MBC for recombinant SpHyastatin was incubated with *S. aureus* as described above. At various time points, 6 μL of the treated bacterial suspension was added to 10 mM NaPB, mixed and spread on nutrition broth agar. The numbers of CFU were determined after incubation at 37°C for 18–24 h. The percentage of CFU was defined relative to the CFU obtained in the control (100% CFU at 0 min).

### Preparation of Polyclonal Antibody against SpHyastatin

Five BALB/C mice raised in the Animal Culture Centre of Xiamen University were immunized with the collected recombinant SpHyastatin to generate the antiserum. The initial immunization was subcutaneously injected with 200 μg of SpHyastatin mixed with Freund’s complete adjuvant. The second injection was carried out after an interval of 2 weeks with 100 μg of SpHyastatin mixed with incomplete form Freund adjuvant. Subsequently, the booster immunizations were given in the same way each week for three times in all. The blood of mice was collected 6 days after the last boost and clotted overnight at 4°C, then centrifuged at 6000 *g* for 10 min at 4°C to obtain the antiserum. The crude serum obtained was purified using a Nab Spin Kit, 1 mL for Antibody Purification (Thermo Scientific, USA) following the manufacturer’s protocol. The titer, purity and specificity of the purified anti-SpHyastatin antibody were analyzed using ELISA, SDS-PAGE and Western blot.

### Binding Properties of SpHyastatin to Lipopolysaccharides and Lipoteichoic Acid

SpHyastatin binding properties to cell wall components, LPS (0127: B8, Sigma, USA) from *E. coli* and LTA (L2515, Sigma, USA) from *S. aureus* were investigated using ELISA modified from a previous method (Zhang et al., 2013). First, 50 μL (3 μg) of LPS or LTA solution (prepared in 100 mM Na_2_CO_3_, 20 mM EDTA, pH 9.6) were coated on a flat bottom 96-well ELISA plate. The content was heated at 60°C for 2 h until the water evaporated completely and the excess bacterial components were then washed out with PBS. The plate was blocked with 5% (w/v) BSA in PBS for 1 h at room temperature prior to incubation with serial dilutions of the purified SpHyastatin (0–0.04 mg/mL, 100 μL/well). The samples were cultured with 100 μL of anti-SpHyastatin antibody (1:1000 dilution, 100 μL/well) for 2 h before incubating with HRP-labeled Goat Anti-Mouse IgG (1:1,000 dilution, 100 μL/well) for 1 h. Subsequently, tetramethylbenzidine (100 μL/well) was added to the plate, which was incubated at room temperature for 15 min. The colorimetric reaction was then terminated by adding 1 M H_2_SO_4_. Finally, the absorbance (450 nm) of each well was detected using a multifunctional microplate reader (TECAN GENios, Switzerland). The assays were made in triplicate. Scatchard plot analysis was used to assess the binding results. The binding parameters, apparent dissociation constant (Kd), and the maximum binding (Amax) were determined using non-linearly fitting as A = Amax [L]/(Kd + [L]), where A is the absorbance at 450 nm and [L] is the protein concentration.

### Chitin-Binding Assay

Ten microgram portions of the recombinant SpHyastatin, PRD or the synthetic CRD were separately used to estimate chitin-binding properties using a modified protocol (Yao et al., 2012). Briefly, each peptide was reconstituted in 50 μL buffer (20 mM Tris-HCl buffer pH 8.0, 20 mM NaCl), and incubated for 2 h at 4°C with 1 mg chitin (Sigma, USA). After a 15 min centrifugation at 4°C, the supernatants containing unbound proteins were recovered and boiled with 4 × SDS-PAGE buffer, while the pellets were washed five more times with 1 mL wash buffer (20 mM Tris-HCl buffer pH8.0, 20 mM NaCl) and then boiled with 1 × SDS-PAGE buffer for analysis of the bound fraction. Finally, the total, unbound and bound samples were subjected to 15% SDS-PAGE and were detected through Western blotting using the anti-SpHyastatin antibody.

### Propidium Iodide (PI) Uptake Assay

The integrity of the microbial membrane treated with SpHyastatin was determined by measuring the influx of PI using a modified method described previously (Wang et al., 2013). Briefly, the tested microbes were grown to mid-logarithmic phase in the appropriate growth media. Aliquots of microbial suspension (10^8^ cfu/mL) were incubated with 100 mM NaPB (blank) or 400 μg/mL SpHyastatin (a concentration of supra-MBC of peptide) at a volume ratio of 1:1 at 30°C for 2 h. The suspension was mixed with PI (100 μg/mL) followed by incubation for 5 min at room temperature in the dark. Fluorescent images were obtained with a multiphoton laser scanning microscope (Zeiss Lsm 780 NLO, Germany).

### SEM Examination of the Microbial Membrane

The ability of SpHyastatin to alter the morphology of microbes was visualized using SEM as described earlier (Wei et al., 2013). Briefly, microbial suspensions were prepared and incubated with SpHyastatin as described in the PI uptake assay. The cells were then fixed with 2.5% glutaraldehyde for 3 h and washed three times with 100 mM NaPB before being placed on a poly-L-lysine coated glass slide at 4°C for 30 min. The samples were subsequently dehydrated with graded ethanol and tert-butyl alcohol followed by freezing at 4°C and lyophilized using the critical point method. Finally, the cells were gold-coated and observed with an FEI XL-30 Environmental Scanning Electron Microscope (USA).

### SpHyastatin Localization Studies

SpHyastatin localization studies were carried out based on a previous report with slight modifications (Morita et al., 2013). Briefly, SpHyastatin was incubated with microbial suspensions as mentioned above. After being fixed with 4% paraformaldehyde for 30 min and washed three times, microbial cells were immobilized on a poly-L-lysine coated glass slide at 4°C for 30 min. The cells were treated with 0.1% Triton X-100 for 10 min and then blocked in 10% goat serum for 1 h at room temperature. After blocking, cells were reacted with primary antibody anti-SpHyastatin overnight at 4°C followed by incubation for 1 h, in the dark, with Dylight 649 conjugated goat anti-mouse IgG (Multisciences, China). Finally, DAPI (Invitrogen, USA) was used for bacterial staining. The fluorescence of cells was observed with a multiphoton laser scanning microscope (Zeiss Lsm 780 NLO, Germany).

### Evaluation of *S. paramamosain* Survival When Challenged with Bacteria Co-treated or Pre-treated with SpHyastatin

To investigate the *in vivo* function of SpHyastatin, we performed mortality comparison using *S. paramamosain* (averaging 40 ± 5 g each) as described previously (Pan et al., 2011). Briefly, both of the recombinant SpHyastatin and the pathogenic bacterium *Vibrio parahaemolyticus* were reconstituted in 1 × PBS for the experiments. For co-treatment, the peptide (10 μg/crab) was incubated with *V. parahaemolyticus* (2 × 10^7^ CFU/crab) at room temperature for 30 min and then injected into the base of the right fourth leg of crabs. On the other hand, the immune protection role of SpHyastatin was further investigated by injecting 10 μg of peptide/crab 1 h before the *V. parahaemolyticus* injection, and the survival rates were recorded.

### Quantification of Immune Gene Expression after Treatment with Various Stimuli

*S. paramamosain* was injected with PBS, *V. parahaemolyticus* (2 × 10^6^ CFU/crab), *V. parahaemolyticus* plus SpHyastatin (10 μg/crab) or SpHyastatin alone. Hemocyte samples were collected at 6, 12, 24, and 48 h post-injection and were utilized to extract total RNA using TRIzol Reagent (Invitrogen, USA). The cDNA was synthesized using a PrimeScript^TM^ RT Reagent Kit (Takara Biotechnology Co., Ltd., Dalian, China). qPCR was carried out to determine the relative expression level of the selected genes in a 7500 Real-Time PCR System (Applied Biosystems, Carlsbad, CA, USA) within a 20 μL reaction volume containing 10 μL of Power SYBR Green PCR Master Mix (Roche, USA), 1 μL cDNA template, 5 pmol of each primer. The PCR reactions were 50°C for 2 min, 95°C for 10 min, then 40 cycles (15 s at 95°C, 1 min at 60°C). β*-actin* (GU992421), a stably expressed reference gene, was quantified to normalize the relative expression levels for selected genes. Specific qPCR products were confirmed by analysis of melting curve. Only primers, listed in Supplementary Table S2, with the amplification efficiencies calculated within 90–110% were selected for evaluating the relative expression profiles using the algorithm of the 2^-ΔΔCt^ method (Arocho et al., 2006).

GenBank accession numbers for the gene sequences used in the present study are as follows: *SpToll*: JQ327142; *ALF2*: JQ069031; *Crustin*: EU161287; *SOD*: FJ774661; *CAT*: FJ774660; *GPx*: JN565286; and *Lysozyme*: HQ158762. The sequences of *Spaetzle*, *Cactus* and *Dorsal* were obtained from a transcriptome of *S. paramamosain* (unpublished data). Three replicates were performed, analyzing at least 5 crabs for each sample. One-way analysis of variance (ANOVA) was used for statistical analysis using SPSS 11.0 to determine the expression difference within groups. Statistically significant levels were accepted at *p* < 0.05.

## Results

### Analysis of the Overall Peptide Structure of SpHyastatin

**FIGURE 1:**
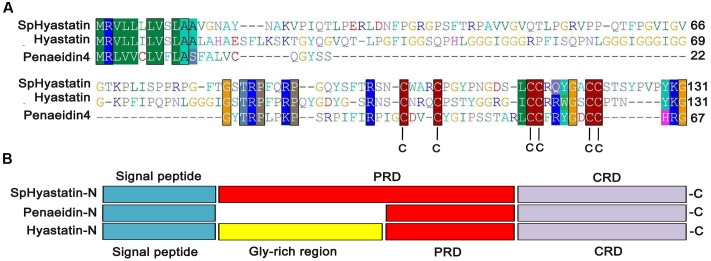
**Characterization of the primary structure property of SpHyastatin.** **(A)** Multiple sequence alignment of SpHyastatin with hyastatin from *H. araneus* (ACQ76432) and penaeidin from *L. vannamei* (ABA55000) using Clustal X 1.83 software. **(B)** Comparison of primary structure of hyastatins and penaeidins. Different colors represent different structure domains.

Multiple sequence alignment (**Figure 1A**) revealed that a short PRP-containing portion (TRPFPRP) located in the N-terminal region was conserved across the amino acid sequences of hyastatins and penaeidins. Six conservation Cys residues located in the C-terminal of hyastatins arrayed almost the same as in the shrimp penaeidins, and the Cys array of these peptides is C1-(X2-3)-C2-(X9-10)-C3-C4-(X5)-C5-C6 (Gueguen et al., 2006). Consequently, the identity of structure property between hyastatins and members of the penaeidin families was noticeable as shown in **Figure 1B**. Each contained a signal peptide and a conserved C-terminal CRD. In addition, SpHyastatin had a prolonged PRD, *H. araneus* hyastatin consisted of a Gly rich region and a short PRD, while penaeidins contained only a short PRD.

### Antimicrobial Activity Assays

As shown in **Supplementary Figures S1A,B**, both SpHyastatin and PRD SpHyastatin were successfully expressed in *P. pastoris*. The antimicrobial and bactericidal activities (shown in the MIC and MBC, respectively) of the recombinant SpHyastatin were examined using a panel of microorganisms. The MIC and MBC values obtained are summarized in **Table 1**. The SpHyastatin protein displayed a broad antimicrobial spectrum, exhibiting potent activities against both Gram-positive (*Micrococcus luteus*, *S. aureus*, *Corynebacterium glutamicum*, *Micrococcus lysodeikticus*) and Gram-negative bacteria (*Pseudomonas stutzeri*, *Pseudomonas fluorescens*, *Aeromonas hydrophila*) with MIC values of 0.63–2.5 μM, as well as values of MBC lower than 10 μM. In addition, SpHyastatin showed antimicrobial activity against *P. pastoris* GS115 with a value of MIC 2.5–5 μM.

**Table 1:** Antimicrobial and bactericidal spectra of SpHyastatin, PRD SpHyastatin and CRD SpHyastatin.

Microorganisms	CGMCC	SpHyastatin		PRD SpHyastatin		CRD SpHyastatin
				
	No.^a^	MIC^c^ (μM)	MBC^c^ (μM)	MIC (μM)	MBC (μM)	MIC (μM)
**Gram-negative bacteria**						
*Escherichia coli*	1.2389	>10	>10	>10	>10	>37
*Vibrio prahaemloyticus*	1.1615	>10	>10	>10	>10	>37
*Vibrio alginolyticus*	1.1833	>10	>10	>10	>10	>37
*Vibrio harvryi*	1.1593	>10	>10	>10	>10	>37
*Vibrio fluvialis*	1.1609	>10	>10	>10	>10	>37
*Pseudomonas stutzeri*	1.1803	0.63–1.25	0.63–1.25	2.5–5	>10	>37
*Pseudomonas fluorescens*	1.0032	1.25–2.5	5–10	2.5–5	2.5–5	25–37
*Aeromonas hydrophila*	1.2017	1.25–2.5	>10	>10	>10	>37
*Shigella flexneri*	1.1868	>10	>10	>10	>10	>37
*Pseudomonas aeruginosa*	1.0205	>10	>10	>10	>10	>37
**Gram-positive bacteria**						
*Bacillus subtilis*	1.108	>10	>10	>10	>10	>37
*Bacillus cereus*	1.447	>10	>10	>10	>10	>37
*Micrococcus luteus*	1.634	1.25–2.5	1.25–2.5	>10	>10	25–37
*Staphylococcus aureus*	1.363	0.63–1.25	0.63–1.25	2.5–5	2.5–5	>37
*Corynebacterium glutamicum*	1.1886	1.25–2.5	5–10	>10	>10	25–37
*Micrococcus lysodeikticus*	1.0634	2.5–5	2.5–5	2.5–5	>10	>37
**Yeast**						
*Candida albicans*	2.2411	>10	>10	>10	>10	NT^d^
*Pichia pastoris* GS115	Invitrogen^b^	2.5–5	5–10	>10	>10	NT


*^a^CGMCC No. China General Microbiological Culture Collection Number.*

*^b^*Pichia pastoris* GS115 was purchased from Invitrogen.*

*^c^The values of MIC and MBC are expressed in this table as the interval [a]-[b], where [a] is the highest concentration tested at which microbial growth can be observed, and [b] the lowest concentration yields no detectable microbial growth or causes 99.9% microorganisms growth inhibition (*n* = 3).*

*^d^NT, not tested.*

The antimicrobial activity of the recombinant PRD and the synthetic CRD was separately measured in comparison with the identical target species spectrum obtained using the full-length SpHyastatin. As shown in **Table 1**, PRD was active in Gram-positive (*S. aureus*, *M. lysodeikticus*) and Gram-negative bacteria (*P. stutzeri*, *P. fluorescens*) with MIC values of 2.5–5 μM. Compared with the antimicrobial activity of the SpHyastatin mature peptide, PRD gave higher or similar MIC values toward the above four strains tested, but was ineffective against the growth of *A. hydrophila*, *C. glutamicum*, *M. luteus*, and *P. pastoris* GS115. CRD inhibited the growth of *P. fluorescens*, *M. luteus*, and *C. glutamicum* only at high concentrations (MIC = 25–37 μM).

### Killing Kinetics of SpHyastatin in *S. aureus*

**FIGURE 2:**
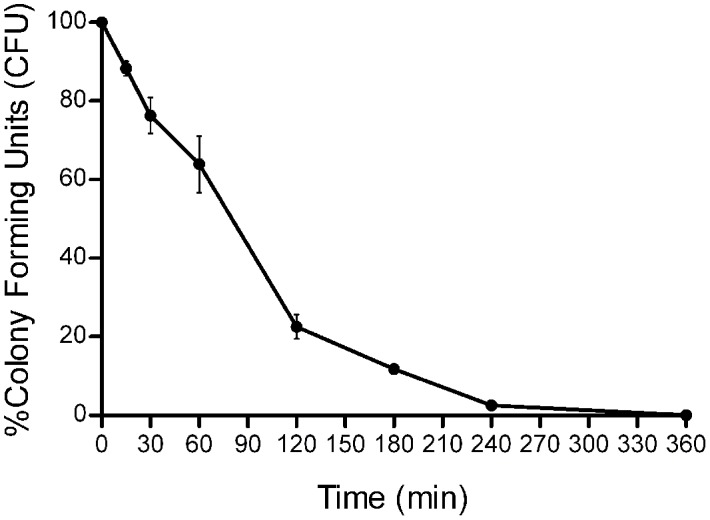
**Killing kinetics of *S. aureus* cells treated with recombinant SpHyastatin.** The percentage of CFU is defined relative to the CFU obtained in the control (100% CFU at 0 min). Each bar represents the means ± SD of three determinations (*n* = 3).

The results of the time-killing kinetic assay employed to further evaluate the bactericidal activity of SpHyastatin using a highly sensitive strain of *S. aureus* are shown in **Figure 2** and reveal that when SpHyastatin was incubated with *S. aureus* at 4 × MBC for different time intervals, it killed 90% of the bacteria after incubation for 4 h and destroyed all the bacteria around 6 h.

### Microbial Associated Molecule-Binding Properties

**FIGURE 3:**
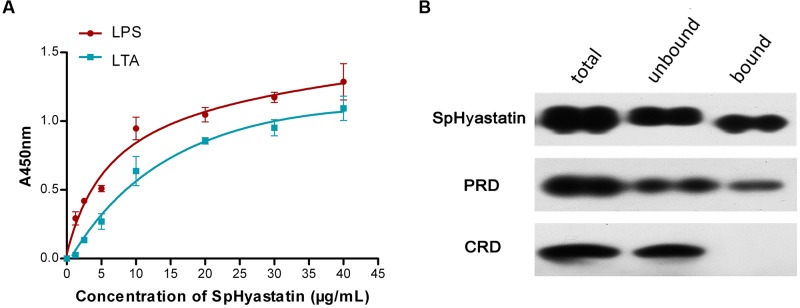
**Binding properties of SpHyastatin to bacterial or fungal associated molecules.** **(A)** The binding affinity of SpHyasatin protein to LPS and LTA tested under ELISA. Each point represents the mean ± SD (*n* = 3). **(B)** Chitin-binding capacity of SpHyastatin, PRD and CRD. The total, unbound and bound fractions were separated using SDS-PAGE and analyzed with Western blot using anti-SpHyastatin as the first antibody.

The binding properties of SpHyastatin protein to different microbial surface molecules as measured using ELISA are shown in **Figure 3A**. SpHyastatin could bind tightly to LPS and LTA, even at a very low concentration of 1.25 μg/mL. It was also found that more SpHyastatin bound to immobilized LPS and LTA as increasing amounts of the protein were added, indicating that the binding of the protein for each molecule was in a concentration dependent manner. Additionally, Scatchard plot analysis showed that SpHyastatin bound to LPS and LTA with apparent dissociation constants (Kd) of 5.58 × 10^-6^ M and 1.32 × 10^-5^ M. This result suggested that SpHyastatin exhibited stronger binding activity with LPS than with LTA. As shown in **Figure 3B**, SpHyastatin was recovered in the bound fractions, indicating that it was able to bind tightly to chitin. PRD alone displayed binding properties similar to the entire molecule. On the contrary, CRD could be detected only in the unbound supernatant, thus denoting that CRD did not exhibit specific binding to chitin.

### The Uptake of PI into Microbes Induced by SpHyastatin

**FIGURE 4:**
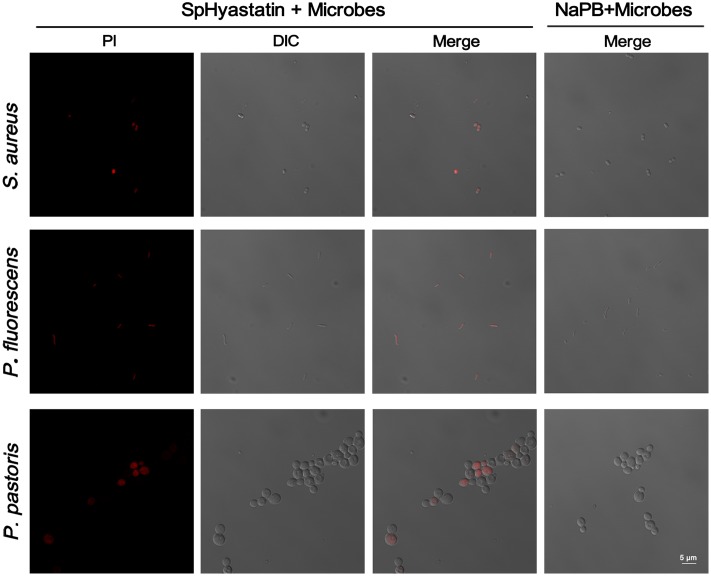
**Effect of SpHyastatin on the integrity of the microbial membrane.** Exponential phase microbial cells suspended in NaPB were incubated for 2 h at room temperature with SpHyastatin. Subsequently, the cells were treated with PI and observed using confocal laser scanning microscopy to analyze the integrity of the microbial membrane.

In order to characterize the bactericidal mechanism of SpHyastatin, a PI uptake assay was utilized in this work. PI, a DNA-intercalating fluorescent dye, is commonly used to evaluate compromised cell membrane permeability. As shown in **Figure 4**, confocal laser scanning microscopy revealed that almost all the microbial cells were fluorescently labeled by PI after 2 h of incubation with SpHyastatin, with no fluorescence being visualized in the control group. That is, SpHyastatin may injure microbial membranes and induce the uptake of PI.

### SpHyastatin Induces Morphological Changes in Microbes

**FIGURE 5:**
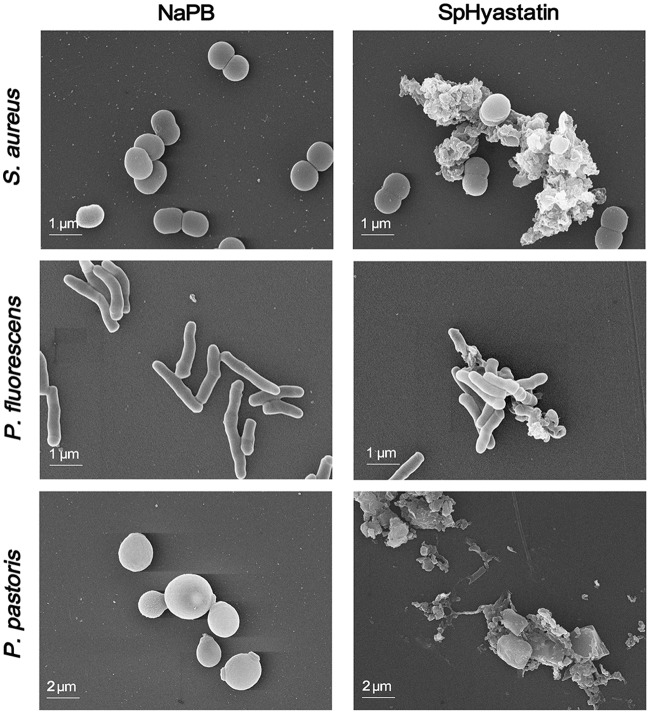
**Effects of SpHyastatin on the membrane morphology of *S. aureus*, *P. fluorescens*, and *P. pastoris* by SEM.** Microbes treated with NaPB (**left column**, control) show a normal smooth surface, while those treated with SpHyastatin (**right column**) reveal clear morphological changes.

In order to gain a better understanding of the interaction between SpHyastatin and microbes, SEM was employed to observe directly the morphological changes in the microbial membrane after exposure to SpHyastatin (**Figure 5**). After SpHyastatin treatment, the SEM images of *S. aureus*, *P. fluorescens*, and *P. pastoris* showed a clearly rougher surface, even the emergence of collapsed architecture, in contrast to the normal and smooth surfaces of the untreated group.

### Different Localization Sites of SpHyastatin on Different Microbes

**FIGURE 6:**
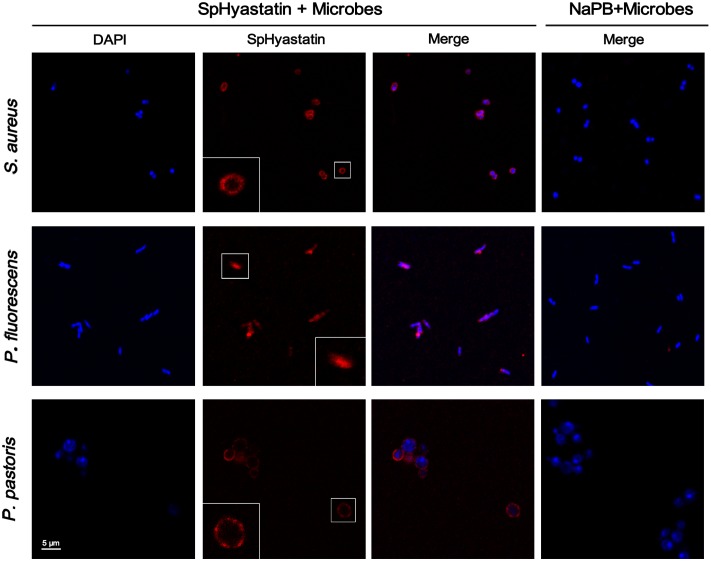
**Localization of SpHyastatin in *S. aureus*, *P. fluorescens*, and *P. pastoris* as seen using confocal laser scanning microscopy.** Aliquots of microbial cells were incubated with SpHyastatin for 2 h at room temperature and then used for immunofluorescence analysis. The cells in squares were magnified images to show details of fluorescence localization. For the untreated cells, only merged images are shown.

Immunofluorescence was used to determine the action site of SpHyastatin against various microbes. Two different localization patterns were observed from the confocal laser scanning microscopy images (**Figure 6**). Fluorescence localization of SpHyastatin pre-treated with *S. aureus* and *P. pastoris* was present around the cellular membrane but absent inside the cell. On the other hand, this peptide was observed localized throughout the cell of *P. fluorescens*, which likely resulted from the peptide penetrating the cell membrane and accumulating in the cytoplasm.

### SpHyastatin Increases *S. paramamosain* Survival in *V. parahaemolyticus* Infection

**FIGURE 7:**
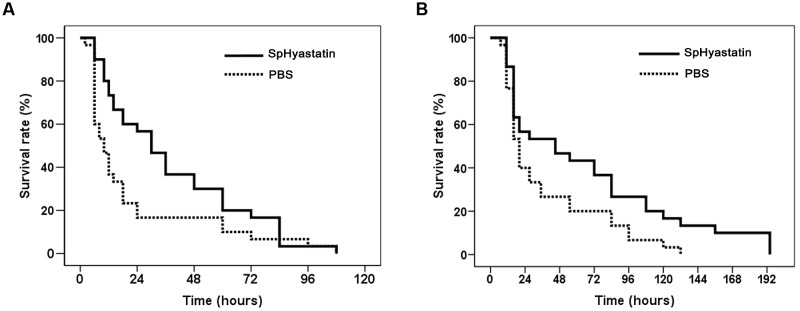
**The ability of SpHyasrtatin to protect *S. paramamosain* from a lethal challenge by *V. parahaemolyticus*.** **(A)** *V. parahaemolyticus* incubated with SpHyasrtatin or PBS for 30 min at room temperature, and then injected into crabs. **(B)** Crabs were injected with SpHyastatin or PBS at 1 h prior to *V. parahaemolyticus* infection (*n* = 30). The survival curve of each experimental group was analyzed using the Kaplan–Meier Log rank test.

In order to evaluate the *in vivo* protective activities of SpHyastatin against *V. parahaemolyticus* infection, co- and pre-treated groups were designed. As shown in **Figure 7A**, SpHyastatin could delay the original infectious process when co-treated with *V. parahaemolyticus*. Especially, the median-lethal time for the peptide co-treatment injected group was 30 h, while that of the PBS co-treatment injected group was 10 h. In addition, *S. paramamosain* pre-treatment with SpHyastatin at 1 h prior to *V. parahaemolyticus* infection exhibited a higher survival rate than those of crabs pre-treated with PBS (*P* < 0.05) (**Figure 7B**).

### SpHyastatin Modulating the *V. parahaemolyticus* Infection-Mediated Immune Gene Expression Profiles in *S. paramamosain*

**FIGURE 8:**
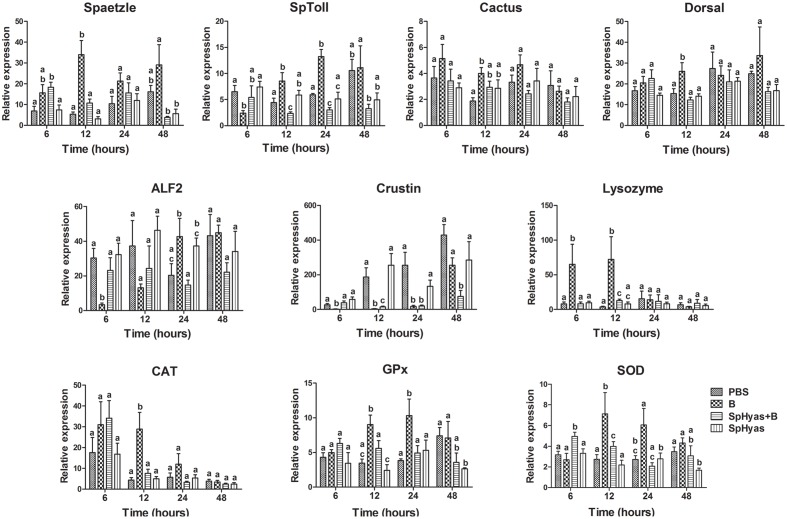
**SpHyastatin effects on the *V. parahaemolyticus* infection-mediated immune gene expression profiles in *S. paramamosain*.** Crabs were divided into PBS, *V. parahaemolyticus* (B), SpHyastatin + *V. parahaemolyticus* (SpHyas + B) and SpHyastatin (SpHyas) groups. The expression levels of *Spatzle*, *SpToll*, *Dorsal*, *Cactus*, *Lysozyme*, *Crustin*, *ALF2*, *SOD*, *CAT*, and *GPx* were evaluated using qPCR at 6, 12, 24, and 48 h post-injection. The results showed that the injection of SpHyastatin into crabs could modulate the mRNA expression of the tested immune or antioxidant-associated genes in *S. paramamosain* with a challenge of *V. parahaemolyticus* at 6, 12, 24, or 48 h post-injection, respectively. In particular, the individual expression level of eight tested genes except for *Cactus* and *ALF2* was significantly changed at 12 h post-injection. Each bar represents the means ± SD (*n* = 5). The same letters (a, b, c, d) indicate no significant difference between groups and different letters indicate statistically significant differences between groups (*P* < 0.05) as calculated by one way ANOVA followed by Tukey test. It was noted that only the means at each time point were compared for the denotation with the letters, whereas the means at different time points could not be compared with one another.

Quantitative real-time PCR revealed the effect of SpHyastatin on the immune response to *V. parahaemolyticus* (**Figure 8**). Compared to the PBS controls, the transcription levels of the canonical component related to the Toll pathway including *Spaetzle*, *SpToll*, *Cactus*, and *Dorsal* were increased at various times during treatment with *V. parahaemolyticus*. However, co-injection of SpHyastatin and *V. parahaemolyticus* obviously attenuated *V. parahaemolyticus* mediated induction of the above. Expression of antioxidant enzymatic genes, such as *CAT*, *SOD*, and *GPx* were significantly decreased by SpHyastatin and *V. parahaemolyticus* co-treatment compared with the bacterially treated group. qPCR analysis further showed that the expression of *Crustin* and *ALF2* were obviously down-regulated in response to pathogen injection, but co-treatment alleviated the pathogen-mediated down-regulation of these two AMPs. On the contrary, the expression of *Lysozyme* was greatly increased by pathogen stimulation, while the enhancement was abrogated in the co-treated group. It was clearly seen that SpHyastatin treatment alone induced hardly any change in the level of gene expression in any group.

## Discussion

In the study, SpHyastatin was characterized for the first time in the marine crab *S. paramamosain*. SpHyastatin possessed both PRD and CRD and shared a certain similarity with *H. araneus* hyastatin (Sperstad et al., 2009) and some shrimp penaeidins (O’Leary and Gross, 2006; Woramongkolchai et al., 2011). These studies revealed that the domain of either PRD or CRD is generally contributed to the antimicrobial activity of an AMP, however, even with the similar domains of PRD and CRD the antimicrobial activity are different among AMPs. The finding of SpHyastatin with multi-domain structure attracted our attention to further explore what antimicrobial feature of SpHyastatin may have and is it unique or similar to that confirmed in other AMPs? The structure-activity studies demonstrated that PRD SpHyastatin alone showed potent antimicrobial activity with MIC values a little higher than SpHyastatin mature peptide, while CRD SpHyastatin displayed a relative weak antimicrobial activity. This meant that PRD together with CRD were indispensable to confer upon SpHyastatin the optimum antimicrobial activity as shown in the studies of *H. araneus* hyastatin (Sperstad et al., 2009), arasin 1 (Paulsen et al., 2013) and shrimp penaeidins (Cuthbertson et al., 2005). These results from structure-activity studies suggested that the PRD SpHyastatin was probably the target active region similar to the previous studies of PRD Pen4 in *Litopenaeus setiferus* and the PRD arasin 1 in *H. araneus*, which maintain a lower but still potent antimicrobial activity in comparison with the full-length peptide (Cuthbertson et al., 2004, 2005; Paulsen et al., 2013). This result was somewhat different from the reported rHyastatin-N-term which is ineffective at concentrations up to 25 μM in all tests (Sperstad et al., 2009), suggesting an association between the structure and function for both AMPs. Similarly the PRD PEN3 from *Litopenaeus vannamei* does not inhibit microorganism growth even at concentrations >50 μM (Cuthbertson et al., 2005, 2008). Apparently, SpHyastatin possessed the unique antimicrobial structure of PRD which differentiated it from the homologous hyastatin of *H. araneus*. The variability in the primary sequence of PRD was likely to result in the different activity of both peptides, since the CRD sequence was totally conserved in both peptides.

The present study further proved that SpHyastatin could bind to various anionic cell wall components such as LPS and LTA present on the surface of Gram-negative and Gram-positive bacteria owing to carrying a net positive charge. The data provided the evidence that electrostatic attraction between SpHyastatin and the bacterial surfaces was crucial for performing antimicrobial activity. The chitin-binding studies showed that PRD, together with the full-length SpHyastatin, had a strong association with chitin (a component of the fungal cell wall) similar to that observed in the rHyastatin-N-term of *H. araneus* (Sperstad et al., 2009). Conversely, CRD SpHyastatin, containing a partial conservation of the chitin-binding motif similar to *Tachypleus tridentatus* tachycitin (Suetake et al., 2002) and *Mytilus coruscus* mytichtin-1 (Qin et al., 2014), did not present a homogeneous chitin-binding property as the penaeidins, whose antifungal activities are probably mediated by their COOH-terminal domain (Destoumieux et al., 2000). Thus, we deduced that the high affinity between PRD SpHyastatin and chitin might be an initial and crucial step for further anti-*P. pastoris* activity.

Among the reported antimicrobial mechanisms of AMPs, one was the disturbance of the integrity of the cell membrane resulting in the release of ions and metabolites and/or lysis of the cell (Reddy et al., 2004; Yan et al., 2013). In our study, SpHyastatin could induce the influx of PI into cells, indicative of a membrane permeabilizing killing action. In addition, SEM results directly presented evidence that SpHyastatin induced morphological changes and eventually led to lysis of both bacteria and fungi, which was in accordance with the results of the PI uptake assays. Further observation using confocal laser scanning microscopy showed that the behavior of SpHyastatin killing *P. fluorescens* was similar to that of the NK-18 derived from mammalian NK-Lysin and NKLP27 from *Cynoglossus semilaevis*, all of which were able to target not only the cell membrane but also the cytoplasm, followed by disturbance of the bacterial membrane of the targeted cells (Hayes et al., 2013; Yan et al., 2013; Zhang et al., 2014). Similarly, the plant AMP NaD1 kills *Candida albicans* and *Fusarium oxysporum* by way of entry into the microbial cytoplasm and membrane permeabilization (van der Weerden et al., 2008; Hayes et al., 2013). Nonetheless, it was interesting to note that SpHyastatin acted only on the cell membrane of *S. aureus* and *P. pastoris* and thus shared a similar cell-killing mechanism to magainin II that kills *E. coli* via permeabilizing the cell membrane without penetrating it (Matsuzaki, 1998). Sphistin, the N-terminus of crab histone H2A, also induces microbial cell death solely by permeabilizing target membrane other than penetrating cell membrane (Chen et al., 2015). Obviously, the binding positions of SpHyastatin on the tested microbial cells were distinct among *S. aureus*, *P. fluorescens* and *P. pastoris*, whereas *P. fluorescens* exhibited a lower degree of cell rupture than *S. aureus* and *P. pastoris* (**Figure 5**). Our present findings indicated that SpHyastatin was likely to exert different cell-killing mechanisms on different species of pathogens, and a similar study reports that tachyplesin I uses different modes of action against *E. coli* and *S. aureus* (Hong et al., 2015). This interesting observation will lead us to further investigate whether an AMP like SpHyastatin could specifically alter its antimicrobial action against different invading pathogens *in vivo* through its morphological adaptation to the different cell surface structure, composition and properties of Gram-negative bacteria, Gram-positive bacteria and fungi. This would be a very important antimicrobial innate defense strategy for marine crabs to maintain homeostasis in a complex environment.

In the present study, the *in vivo* function of SpHyastatin was characterized through bacterial challenge experiments. As expected, the recombinant SpHyastatin was capable of improving the survival rate of crabs against the deadly pathogen *V. parahaemolyticus*, suggesting that this peptide had a resistant property similar to hepcidin (TH) 2–3, hepcidin 1–5 and epinecidin-1. These marine-derived AMPs are able to enhance the survival of mice or fish to different pathogenic challenges (Wang et al., 2010; Pan et al., 2011, 2012; Huang et al., 2013). The potential medicinal application of marine-derived bioactive peptides as therapeutic agents for both human and animal welfare is attracting much attention (Ngo et al., 2012; Nasri and Nasri, 2013; Jain et al., 2015). Our current study presented evidence that SpHyastatin was more likely to be a potential prophylactic agent, which could be used in aquaculture and veterinary medicine.

As observed, SpHyastatin importantly conferred immune protection against pathogenic infection in *S. paramamosain* (**Figure 7**). In our study, we therefore further investigated the immune effect of SpHyastatin on pathogenic infection. As a result, we found that SpHyastatin could alleviate *V. parahaemolyticus*-induced changes in the transcription levels of both immune-associated genes (*Spatzle*, *SpToll*, *Dorsal*, *Cactus*, *Lysozyme*, *Crustin*, and *ALF2*) and antioxidant-associated genes (*SOD*, *CAT*, and *GPx*). As evidenced by the *in vitro* binding activities to LPS and LTA in our study, SpHyastatin might be an endotoxin-neutralizing protein that may contribute to interfere with the initiation of LPS or LTA mediated signal transduction, alter the expression profiles of immune mediators, and dampen the potentially harmful pro-inflammatory responses such as cationic peptide LL-37 (Hirata et al., 1994; Scott et al., 2002). Such anti-inflammatory properties are demonstrated in several other marine-derived AMPs, for example, shrimp and *Limulus* anti-lipopolysaccharide factor (Battafaraono et al., 1995; Lin et al., 2013), and *Strongylocentrotus droebachiensis* centrocin 1 (Bjorn et al., 2012) inhibit the secretion of pro-inflammatory cytokines on mammalian hosts. It was further interesting to note that the cumulative mortality in crabs pre-treated with SpHyastatin and bacterium was significantly decreased. The reason was likely to be due to its anti-inflammatory activities while not associated with its bacteriostatic property, since SpHyastatin was not able to inhibit the growth of *V. parahaemolyticus in vitro*.

In summary, the newly identified SpHyastatin in *S. paramamosain* had potent antimicrobial activity. This peptide can effectively kill microbes via a permeabilizing mechanism and probably had different antimicrobial active modes against different species of bacteria. The recombinant SpHyastatin was likely to confer immune protective resistance against pathogenic challenge in *S. paramamosain* with less causing significant change in level of the mRNA expression of all tested immune and antioxidant-associated genes. SpHyastatin thus possessed some unique antimicrobial features in comparison with other known AMPs in crabs.

## Author Contributions

KW and ZS conceived and designed as well as analyzed the experiments. ZS performed almost the experiments and wrote the paper. KZ, JL, XM, SW, and KQ performed the experiments shown in **Figures 7** and **8**. HP, BC, and FC provided technical assistance and contributed to the preparation of the figures. KW also contributed all of reagents/materials/analysis tools and wrote the paper. All authors reviewed the results and approved the final version of the manuscript.

## Conflict of Interest Statement

The authors declare that the research was conducted in the absence of any commercial or financial relationships that could be construed as a potential conflict of interest.

## Footnotes

1 http://aps.unmc.edu/AP/main.php

## Supplementary Material

**FIGURE S1 | SDS-PAGE analysis of the purified SpHyastatin and PRD SpHyastatin.** **(A)** The purification steps of SpHyastatin. Lane M, molecular weight marker; lane 1, sample taken from the culture of GS115/pPIC9K at 24 h (negative control); lane 2, fusion protein scygonadin/SpHyastatin expressed in *P. pastoris* after induction by methanol for 24 h; lane 3, fusion protein after purification; lane 4, fusion peptide cleaved by enterokinase; lane 5, flow through fractions; lane 6, purified SpHyastatin. **(B)** The purification steps of PRD SpHyastatin. Lane M, molecular weight marker; lane 1, sample taken from the culture of GS115/pPIC9K at 24 h (negative control); lane 2, fusion protein scygonadin/PRD SpHyastatin expressed in *P. pastoris* after induction by methanol for 24 h; lane 3, fusion protein after purification; lane 4, fusion peptide cleaved by enterokinase; lane 5, flow through fractions; lane 6, purified PRD SpHyastatin.
