# Supplementary figures and images for: The New Antimicrobial Peptide SpHyastatin from the Mud Crab Scylla paramamosain with Multiple Antimicrobial Mechanisms and High Effect on Bacterial Infection
Source: Front Microbiol. 2016 Jul 21;7:1140. doi: 10.3389/fmicb.2016.01140 (PMC4954822; doi:10.3389/fmicb.2016.01140)

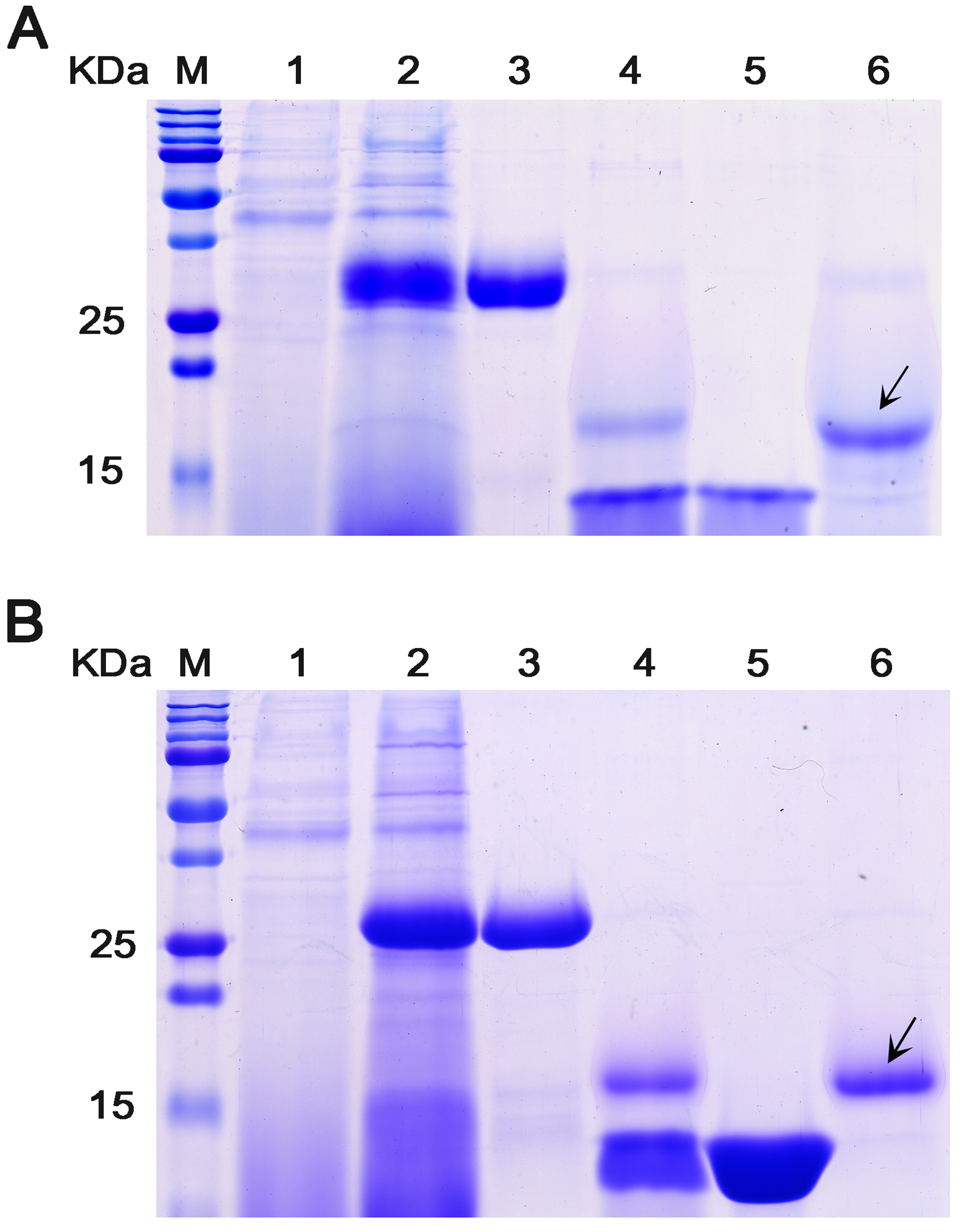

Supplement: Supplementary file 1 [file Image_1.TIF]
