# Supplementary material for: The New Antimicrobial Peptide SpHyastatin from the Mud Crab Scylla paramamosain with Multiple Antimicrobial Mechanisms and High Effect on Bacterial Infection
Source: Front Microbiol. 2016 Jul 21;7:1140. doi: 10.3389/fmicb.2016.01140 (PMC4954822; doi:10.3389/fmicb.2016.01140)
Supplement: Supplementary file 2 [file Table_1.DOCX]

**Table S1**

Microbial strains used in the present study.

|  | Microorganisms |
| --- | --- |
| Gram-negative bacteria | *Escherichia coli, Vibrio prahaemloyticus, Vibrio alginolyticus, Vibrio harvryi, Vibrio fluvialis, Pseudomonas stutzeri, Pseudomonas fluorescens, Aeromonas hydrophila, Shigella flexneri, Pseudomonas aeruginosa* |
| Gram-positive bacteria | *Bacillus subtilis, Bacillus cereus, Micrococcus luteus, Staphylococcus aureus, Corynebacterium glutamicum, Micrococcus lysodeikticus Fleming* |
| Yeast | *Candida albicans, Pichia pastoris* GS115 |
