# Supplementary material for: The New Antimicrobial Peptide SpHyastatin from the Mud Crab Scylla paramamosain with Multiple Antimicrobial Mechanisms and High Effect on Bacterial Infection
Source: Front Microbiol. 2016 Jul 21;7:1140. doi: 10.3389/fmicb.2016.01140 (PMC4954822; doi:10.3389/fmicb.2016.01140)
Supplement: Supplementary file 3 [file Table_2.DOCX]

**Table S2**

Oligonucleotide primers used in the present study.

Bold nucleotides represent the locations of restricted endonucleases.
